# Effects of monensin, phytogenic compounds, or a blend of feed additives on health and performance of dairy cows

**DOI:** 10.1093/tas/txaf140

**Published:** 2025-10-17

**Authors:** Guilherme Poletti, Nathália T S Grigoletto, Caio S Takiya, Alanne T Nunes, Victor V de Carvalho, Cristina S Cortinhas, Tyler D Turner, Francisco P Rennó

**Affiliations:** Department of Animal Production and Animal Nutrition, University of Sao Paulo, Pirassununga 13635-900, Brazil; Department of Animal Production and Animal Nutrition, University of Sao Paulo, Pirassununga 13635-900, Brazil; Department of Animal Production and Animal Nutrition, University of Sao Paulo, Pirassununga 13635-900, Brazil; Academic Department of Agrarian Sciences, Federal University of Technology—Paraná, Pato Branco 85.503-390, Brazil; Deparment of Veterinary Medicine, School of Animal Science and Food Engineering (FZEA), University of São Paulo, Pirassununga 13635-900, Brazil; Department of Innovation & Applied Science, DSM-Firmenich, Nutritional Products, São Paulo, SP 04543-907, Brazil; Department of Innovation & Applied Science, DSM-Firmenich, Nutritional Products, São Paulo, SP 04543-907, Brazil; DSM-Firmenich, Nutritional Products, Brøndby 2605, Denmark; Department of Animal Production and Animal Nutrition, University of Sao Paulo, Pirassununga 13635-900, Brazil

**Keywords:** autolyzed yeast, carbo-aminophosphochelate, organic micromineral, *Saccharomyces cerevisiae*

## Abstract

The objective of this study was to compare the effects of phytogenic additives and monensin on feed intake and digestibility, performance, ruminal fermentation, blood parameters, and nitrogen balance in confined dairy cows. In addition, we aimed to evaluate whether combining phytogenic additives with autolyzed yeast and organic microminerals would produce a synergistic effect on these parameters. Thirty-nine Holstein cows (155 ± 68 d in milk, 35.3 ± 3.31 kg/d milk yield, 644 ± 85.6 kg body weight) were blocked (*n* = 13) by parity, days in milk, and milk yield, and were randomly assigned to one of three treatments: (i) monensin (MON) provided at 17.38 mg/kg DM, (ii) phytogenic additive (PHY), provided at 89.10 mg/kg dry matter (DM), and (iii) a blend of phytogenic additives, autolyzed yeast, and additional organic microminerals (BLD), providing 89.10 mg of phytogenic additive/kg DM, 668 mg of autolyzed yeast/kg DM, and 1.11 g/kg DM of microminerals supplied as carbo-aminophosphochelates. Treatments were administered for 9 wk. Orthogonal contrasts were used to test MON vs. PHY+BLD and PHY vs. BLD. Cows fed phytogenic additives (PHY and BLD) had lower (*P *= 0.038) DM intake compared with those fed MON, without differences in nutrient digestibility and ruminal fermentation parameters (pH, NH_3_-N, or volatile fatty acid proportions). Phytogenic additives (PHY and BLD) also improved feed efficiency (*P *= 0.044), energy-corrected milk yield (*P *= 0.047), and milk fat concentration (*P *= 0.003) compared with MON. Cows receiving MON had higher (*P *= 0.008) serum urea concentrations than those fed phytogenic additives, whereas serum haptoglobin concentrations were lower (*P *= 0.019) in PHY and BLD compared with MON. Nitrogen excretion (g/d) via urine and feces were not affected by treatments. Performance, nutrient intake and digestibility, and ruminal fermentation were similar between cows fed PHY than those fed BLD. These findings indicate that phytogenic additives can replace monensin while improving energy-corrected milk yield and reducing pro-inflammatory biomarkers.

## Teaser

Plant-based additives provide a natural and effective approach to improve dairy cow performance, while also offering environmental advantages

## Introduction

Ionophore antibiotics, such as sodium monensin, are commonly incorporated into ruminant diets to improve feed efficiency by reducing energy and nitrogen losses (McGuffey 2017). This is achieved through selective inhibition of specific rumen microorganisms, which increases the propionate-to-­acetate ratio and reduces ammonia and methane emissions (Duffield et al. 2008; Guan et al. 2006; Kobayashi 2010). Although ionophores such as monensin are not used in human medicine, concerns have been raised that their use may contribute to the development or co-selection of resistance to medically important antibiotics. Recent studies have reported that *Staphylococcus aureus* mutants resistant to monensin exhibit increased virulence (Thomas et al. 2023), and that ionophore resistance genes (*narA* and *narB*) are globally distributed and frequently linked to resistance determinants for human-relevant antibiotics (Li et al. 2023). A broader review has also highlighted the need to further assess potential risks of ionophore use in livestock for antimicrobial resistance development (Dolliver and Gupta 2019).

Phytogenic compounds, such as essential oils and pungent plant-derived compounds, have gained attention as promising alternatives to antibiotics in dairy cow nutrition due to their potential to support rumen function, nutrient absorption, and immunity in cattle (Oh et al. 2017; Rodrigues et al. 2019; Yang et al. 2015). These compounds can selectively reduce harmful microorganisms by disrupting bacterial cell membranes, fostering a healthier rumen environment, and encouraging the growth of beneficial microbes (Helander et al. 1998). In addition, phytogenic compounds present anti-inflammatory, immunomodulatory, antioxidant, and metabolic regulatory properties (Adetunji et al. 2025; Fernandez-Panchon et al. 2008). Among them, L-menthol, thymol, eugenol, and mint oil stand out for their significant benefits, including modulating ruminal fermentation, increasing rumination, modulating salivary proteins, modulating rumen pH, improving immune responses, reducing inflammation, enhancing gut barrier integrity and boosting energy metabolism in cattle (Castillo-Lopez 2023; Kröger et al. 2017; Ramirez et al. 2020; Rivera-Chacon et al. 2022; Yang et al. 2023).

Autolyzed yeast, derived from *Saccharomyces cerevisiae*, is widely used in dairy nutrition because of its benefits to rumen health, including the release of bioactive compounds such as β-glucans, mannoproteins, and amino acids that enhance rumen fermentation, stabilize pH, and improve nutrient digestibility, besides positively influencing immune response (Adili et al. 2020; Kröger et al. 2017; Volman et al. 2008). These compounds promote digestive efficiency, reduce the risk of ruminal acidosis, and may help inhibit pathogen colonization of the gastrointestinal tract (Baker et al. 2022; Takiya et al. 2024; Shurson 2018). Organic microminerals also play an essential role in dairy cattle nutrition due to their high bioavailability compared to inorganic forms, which supports enzymatic processes, immune function, gut barrier integrity, and overall health in lactating cows (Caldera et al. 2019; Goff 2018; Silva et al. 2023; Weiss 2017; Zhang et al. 2020). The combination of phytogenic additives and autolyzed yeast may provide a synergistic effect: phytogenics and autolyzed yeast contribute to microbial modulation, enhancing rumen fermentation and gut health (Humer et al. 2018; Neubauer et al. 2018), while organic microminerals further support enzymatic activity and maintain rumen health by avoiding undesirable interactions often associated with inorganic minerals (Nocek et al. 2006). Although a recent study by Martins et al. (2023) found that while this combination had no significant effect on lactational performance, digestibility, blood cell counts, or energy metabolism markers, it did produce an additive effect on urinary urea nitrogen excretion. The limited literature on this potential ­synergy underscores the need for further research to fully understand and validate these combined effects.

This study hypothesized that supplementation with phytogenic compounds would positively influence milk yield and digestive parameters in lactating cows, resulting in improved performance compared with monensin. Additionally, we hypothesized that combining phytogenic compounds with autolyzed yeast and additional organic microminerals would exert a synergistic effect on cow performance. The objective of this study was to evaluate the effects of these supplements on DM and nutrient intake, apparent total-tract digestibility, feed particle sorting, milk yield and composition, ruminal fermentation, blood metabolites, and nitrogen balance in dairy cows.

## Materials and methods

This study was carried out at the Laboratório de Pesquisa em Bovinos de Leite—LPBL (Laboratory of Dairy Cattle Research; Pirassununga, Brazil) under the approval of the Ethics Committee on Animal Use from the School of Veterinary Medicine and Animal Sciences, University of Sao Paulo (protocol #6276230123).

### Treatments and experimental design

Thirty-nine Holstein cows (155 ± 68 d in milk, 35.3 ± 3.31 kg/d milk yield, 644 ± 85.6 kg BW; 15 primiparous and 24 multiparous) were grouped into 13 blocks (one cow per treatment per block) based on parity, days in milk, and milk yield, resulting in 13 repetitions per treatment. Within each block, cows were randomly assigned to one of the following treatments: (i) monensin (MON) fed at 17.38 mg/kg dry matter (DM) (Rumensin 200, Elanco, São Paulo, Brazil), (ii) phytogenic additive (PHY), fed at 89.10 mg/kg diet DM (Digestarom, dsm-firmenich, Inzersdorf-Getzersdorf, Austria), and (iii) blend of phytogenic additives, autolyzed yeast, and additional organic microminerals (BLD) fed at 1.87 g/kg diet DM, providing 89.10 mg/kg diet DM of the phytogenic additive, 668.54 mg/kg diet DM of autolyzed yeast, 1.5 mg/kg Cu, 3.7 mg/kg Mn, 0.093 mg/kg Se, and 7.41 mg/kg Zn. According to the manufacturers, the phytogenic feed additive in powder form is based on a blend of spices, extracts and herbs including L-menthol, thymol, eugenol, mint oil (Mentha arvensis) and cloves powder (Syzygium aromaticum). The BLD is composed of PHY, autolyzed spray-dried yeast (*Saccharomyces cerevisiae*- Levabon Rumen E, dsm-firmenich, Grenzach-Wyhlen, Germany) and organic microminerals (Cu, Mn, Se, and Zn) provided as carbo-amino-phospho-chelates (Tortuga minerals, dsm-firmenich, São Paulo, Brazil). Additives were incorporated into the concentrate of a TMR. Cows were fed a basal TMR diet (Table 1) formulated according to nutrient requirements estimates established by NASEM (2021) with a 48:52 roughage: concentrate ratio. The mineral mix (Bovigold, dsm-firmenich) included organic microminerals supplied as carbo-amino-phospho-chelates. Consequently, cows in the BLD treatment received a greater amount of organic microminerals compared with those in the MON and PHY treatments. Cows underwent a 2-wk covariate period during which they were fed a basal diet without additives (Table 1). Data on feed intake, apparent total-tract digestibility, milk yield and composition, serum metabolites, and nitrogen utilization were collected during the last 5 days of this period to be included as covariates in the statistical model. After the covariate period, treatments were provided for 9 wk.

**Table 1.** Ingredients and chemical composition of the experimental diet.

Item	TMR
**Ingredient, % diet DM**	
** Corn silage**	48.0
** Ground corn**	18.5
** Soybean meal**	12.7
** Whole raw soybean**	6.40
** Citrus pulp**	8.20
** Dried distillers’ grains with solubles, 45% crude protein**	3.30
**Urea**	0.20
** Sodium Bicarbonate**	0.82
** Limestone**	0.20
**Salt**	0.22
** Minerals and vitamins premix^a^**	1.50
**Chemical, % DM**	
** Dry matter**	49.1 ± 2.15
** Organic matter**	93.3 ± 0.23
**Starch**	25.2 ± 0.43
** Crude protein**	16.5 ± 0.98
** Ether extract**	3.42 ± 0.22
** Neutral detergent fiber (NDF)**	39.0 ± 1.35
** Acid detergent fiber**	23.4 ± 1.11
** Indigestible NDF**	11.5 ± 0.51
**Particle size distribution, % as-fed**	
** >19.0 mm**	8.00 ± 3.85
** 19.0–8.00 mm**	37.1 ± 2.73
** 8.00–4.00 mm**	17.5 ± 5.17
** < 4.00 mm**	37.5 ± 8.01

a Contained per kg: 240 g Ca, 40 g P, 20 g S, 25 g Mg, 10 g K, 70 g Na, 15 mg Co, 7,500 mg Cu, 40 mg I, 20 mg Cr, 2,000 mg Mn, 22 mg Se, 2850 mg Zn, 35,0000 IU Vit A, 10,0000 IU Vit D3, 2,000 IU Vit E.

### Housing, management, and feed analysis

Cows were housed in an open-sided barn equipped with fans and individual pens (17.5 m^2^ of area) containing individual feed bunks and sand beds, and one waterer for every two cows. The average daily temperature in the barn during the experiment was 23.8 ± 1.67°C, with a mean relative humidity of 58.8% ± 6.14% and an average temperature-humidity index of 71.5 ± 2.38. The total mixed ration (TMR) was provided twice daily at 0700 and 1300 h in equal amounts targeting refusals between 5 and 10% (on as-fed basis). Corn silage DM content was monitored thrice per wk to make dietary adjustments when necessary. Silage samples were collected daily throughout each wk of the experiment and pooled into a sample per wk. Ingredients were sampled during the second wk of the covariate period, and at weeks 3, 6, and 9 during the concentrate manufacturing process at the feed mill. Feed samples were analyzed for DM (method 930.15), ash (method 942.05), organic matter (OM; calculated as DM minus ash), crude protein (CP; N × 6.25; method 984.13), and ether extract (EE, method 920.39), according to AOAC International (2000). Neutral detergent fiber (NDF) was analyzed with the addition of alpha-amylase and sodium sulfite (Van Soest et al. 1991), and acid detergent fiber (ADF) and lignin (method 973.18) were assessed according to AOAC International (2000). Fiber analyses were performed using a fiber analyzer (TE-149, Tecnal Equipamentos para Laboratório, Piracicaba Brazil). The starch content in the feed was measured using an enzymatic degradation method (Amyloglucosidase; Novozymes Latin America Ltda., Araucaria, Brazil), as described by Hendrix (1993).

### Nutrient intake, feed sorting index, and apparent digestibility

The feed offered and refused were recorded, and refusals from each cow were collected daily and pooled per wk. All samples were stored at −20°C until chemical analysis. Total mixed ration and refusal samples were collected over two consecutive days during the covariate period and wk 3, 6, and 9 to evaluate the particle size distribution using a Penn State particle separator, following the method of Maulfair and Heinrichs (2012). The feed sorting index was calculated based on the predicted intake of the particle size distribution of TMR and refusals, as described by Silveira et al. (2007). A sorting index of 1 indicates no sorting, values less than 1 indicate sorting against particles of that size, and values greater than 1 indicate sorting for particles of that size.

Fecal samples were collected directly from the rectum over three consecutive d at 9-h intervals (8 samples) during wk 3, 6, and 9. Samples were pooled by cow per wk, and frozen until chemical analysis. Fecal samples were dried at 55°C with forced ventilation for 5 days and ground in a rotary knife mill to pass through 2 and 1 mm-sieves. Samples of feed and feces ground to 2 mm were placed in non-woven tissue bags (5 × 5 cm, 20 mg/cm^2^) and incubated in the rumen of two cannulated Holstein cows on the basal diet without additives for 288 h (Casali et al. 2008). After removal, the bags were rinsed with running tap water until they were clear. Residues were analyzed for NDF, as previously described, to determine the indigestible (iNDF) content. Daily fecal excretion of DM was estimated by dividing iNDF fecal concentration by iNDF intake (Zanferari et al. 2025). Fecal samples were analyzed for OM, CP, NDF, and EE to assess nutrient excretion. Apparent total-tract apparent digestibility was calculated based on nutrient intake and fecal nutrient excretion (Zanferari et al. 2025).

Cows were weighed weekly on two consecutive days after the morning milking and before feeding (0630 h). Body condition scores (BCS) were recorded at the same time as BW measurements (Edmonson et al. 1989).

### Ruminal fermentation

Ruminal fluid samples were collected via an esophageal probe from 12 cows (4 blocks) 4 h after morning feeding, discarding the first 250 mL collected to avoid saliva or mucous contamination. Ruminal fluid pH was measured using a glass electrode and a reference electrode (MB-10, Marte Científica, Santa Rita do Sapucaí, Brazil). Ruminal fluid samples were centrifuged (2000 × *g* for 15 min at room temperature), and 1.8 mL of the supernatant was pipetted into a centrifuge tube containing 400 μL orthophosphoric acid solution (1 *N*) for further volatile fatty acid (VFA) analysis. Peaks of VFA were measured on a gas chromatograph (Shimadzu GC-2010 Plus, Shimadzu, Tokyo, Japan) equipped with an automatic injector (AOC-20i, Stabilwax-DA 30 m capillary column, 0.25 mm ID, 0.25 μm df; Restek, Bellefonte, PA) and a flame ionization detector, as described by Del Valle et al. (2018). Another aliquot of the supernatant (800 μL) was mixed with 400 μL of 1 *N* sulfuric acid solution for NH_3_-N determination using the phenol-hypochlorite method (Broderick and Kang 1980), and the absorbance was measured on a microplate reader (Biochrom Asys, Biochrom, Cambridge, UK).

### Milk yield and composition, and serum metabolites

Cows were milked twice daily (0600 and 1700 h) in a double eight herringbone milking parlor with electronic milk meters (DeLaval, Tumba, Sweden). Milk yield was electronically recorded for each milking shift (Delpro, DeLaval). Milk samples were collected weekly on three consecutive d at each milking during the covariate period and wk 3, 6, and 9. Samples were pooled by day according to morning and afternoon milking. Milk samples were analyzed for concentrations of true protein, fat, and lactose using mid-infrared method (Lactoscan, Entelbra, Londrina, Brazil). Milk yield was corrected for 3.5% fat according to Sklan et al. (1992), as follows: 3.5% FCM = (0.432 + 0.165 × milk fat %) × milk yield (kg/d). Milk yield was also corrected for energy according to NRC (2001). Milk samples were deproteinized with trichloroacetic acid solution (25%; 2:1 vol/vol; Shahani and Sommer 1951) and stored at −20°C until further analysis. Milk urea nitrogen (MUN) concentration was determined using a commercial kit (K-056; Bioclin, Belo Horizonte, Brazil). Somatic cells in milk were analyzed by flow cytometry (Somacount 300; Bentley Instruments, Chaska, MN) in a commercial laboratory (Clínica do Leite, Piracicaba, Brazil). Somatic cell linear score (SCS) was calculated according to Shook (1993).

Blood samples were collected by venipuncture of the tail vessels into lithium heparin vacuum tubes (BD Vacutainer Heparin Tubes, BD, Franklin Lakes, NJ) 4 h after the morning feeding at wk 3, 6, and 9. Two separate samples were obtained: one for immediate analysis using portable devices and another for serum preparation and subsequent laboratory analyses. The first sample was analyzed directly for concentrations of electrolytes (e.g., Na, K, Cl), metabolites (e.g., total protein, albumin, globulin), blood gases, and other critical parameters (e.g., pH, hematocrit, extracellular base excess, anion GAP) using a VetScan Chemistry Analyzer (Zoetis; Parsippany, NJ) and the i-STAT portable clinical analyzer (Abbott Point of Care, Windsor, NJ), as described by McGovern et al. (2024) and Bardell et al. (2017), respectively. Additionally, blood concentrations of vitamin E and β-carotene were measured using iCheck devices (BioAnalyt, Teltow, Germany), based on fluorescence for vitamin E and photometric color reactions for β-carotene, following the method described by Ghaffari et al. (2019). The second sample was centrifuged (15 min, 2000 × *g*, at room temperature), and the serum was stored at −20°C for further analyses. Glucose, urea, and alanine aminotransferase (ALT) were quantified using commercial kits (Bioclin) via enzymatic colorimetric methods on an automated biochemical analyzer (SBA-200, Celm, Barueri, Brazil). Serum haptoglobin levels were determined in a commercial laboratory (VidaVet, Botucatu, Brazil) following the method of Cooke and Arthington (2013).

### Nitrogen output and excretion of purine derivatives

Urine samples were collected in 500 mL plastic vials after stimulating urination by massaging the area below the vulva. Samples were collected concurrently with feces and pooled as previously described. Urine was analyzed for creatinine and nitrogen concentrations to estimate daily urine volume and nitrogen excretion. Urine creatinine concentration was measured using a commercial kit (Kinetic Creatinine K-067; Bioclin), and absorbance was measured using a spectrophotometer (SBA 200, Celm). Daily urine output was estimated based on a daily creatinine excretion rate of 29 mg/kg BW (Valadares et al. 1999).

Urine samples were diluted at a 1:4 (vol/vol) ratio with 0.036 N sulfuric acid to stabilize purine derivatives (Chen and Gomes 1992). The diluted samples were analyzed for allantoin concentration using the colorimetric method described by Chen and Gomes (1992), and uric acid concentration was measured with a commercial kit (Uric Acid Liquid Stable K-052, Bioclin). The daily urinary excretion of purine derivatives (allantoin and uric acid) serves as an indicator of the microbial protein supply to the rumen (Chen and Gomes 1992).

Milk nitrogen excretion was calculated by multiplying the protein concentration in milk by milk yield and then dividing by 6.38. Fecal nitrogen excretion was determined by multiplying the CP concentration in feces by DM excretion and dividing by 6.25. Unaccounted nitrogen was calculated as: N intake – (milk N excretion + urine N excretion + fecal N excretion), expressed in g/d (Martins et al. 2023).

### Statistical analysis

Data were analyzed using the MIXED procedure in SAS (version 9.4, SAS Institute, Cary, NC), modeling the fixed effects of treatment, time, and their interaction, along with the random effect of block. Each variable included its own pre-trial covariate as a continuous variable with covariate measures incorporated as fixed effects. Cow was defined as the subject in repeated-measures analysis. Multiple covariance structures [CS, CSH, AR(1), ARH(1), TOEP, TOEPH, and UN] were tested, with the best-fitting structure selected based on Bayesian Information Criterion (BIC) values. The Shapiro-Wilk test was used to assess the normality of residuals. Means were adjusted using the LSMEANS procedure, and treatment differences were evaluated through orthogonal contrasts to assess the effects of phytogenic additive supplementation (C1: MON vs. PHY and BLD) and the interaction among phytogenic additives, yeast, and microminerals (C2: PHY vs. BLD). Significance was declared when *P *≤ 0.05 and tendencies when *P *> 0.05 and ≤0.10.

## Results

Phytogenic additive supplementation (PHY and BLD) reduced DM intake by 1.2 kg/d (*P *= 0.038) and lowered OM (*P *= 0.035), NDF (*P *= 0.017), and CP intake (*P *= 0.049) compared with MON (Table 2). An interaction between treatment and time was observed for DM intake (*P *= 0.028), with cows fed the phytogenic additive exhibiting lower intake than those fed MON from weeks 5 to 9 of treatment. Cows fed MON avoided long particles >19 mm (*P *= 0.006), preferentially selected fine particles <4 mm (*P *= 0.037), and tended to select particles between 8 and 4 mm (*P *= 0.052) relative to PHY treatments (PHY and BLD). Within phytogenic treatments, cows fed PHY tended to avoid medium particles (19–8 mm) compared with BLD (*P *= 0.068). Body weight was greater in MON than in PHY and BLD (658 vs. 649 kg; *P *= 0.005), whereas BCS remained unaffected (P ≥ 0.260). Apparent total-tract digestibility of DM, OM, CP, NDF, and EE did not differ among treatments (Table 2). Likewise, ruminal pH, NH_3_-N, and VFA profiles were similar across treatments (*P *≥ 0.198; Table 3).

**Table 2.** Nutrient intake, feed particles sorting index, and apparent total-tract digestibility in lactating dairy cows fed phytogenic additives alone or combined with autolyzed yeast and organic microminerals (*n* = 39 cows, or 13 cows per treatment).

Item	Treatment^a^			SEM	*P-*value^b^	
	MON	PHY	BLD		C1	C2
**Intake, kg/d**						
** Dry matter**	27.9	26.6	26.7	0.47	0.038	0.830
** Organic matter**	26.0	24.7	24.9	0.45	0.035	0.826
** Neutral detergent fiber**	10.4	9.85	9.92	0.182	0.017	0.794
**Starch**	6.62	6.38	6.45	0.115	0.157	0.688
** Crude protein**	4.61	4.40	4.43	0.077	0.049	0.808
** Ether extract**	1.04	1.00	1.01	0.018	0.182	0.591
**Sorting index** ^c^						
** >19.0 mm**	0.878	0.916	0.912	0.013	0.006	0.781
** 19.0-8.00 mm**	0.987	0.984	0.990	0.002	0.918	0.068
** 8.00-4.00 mm**	1.014	1.008	1.005	0.003	0.052	0.372
** <4.00 mm**	1.031	1.024	1.020	0.004	0.037	0.417
**Digestibility, %**						
** Dry matter**	69.7	69.4	69.8	0.60	0.834	0.621
** Organic matter**	70.7	70.4	70.9	0.63	0.945	0.584
** Neutral detergent fiber**	45.1	45.8	45.9	1.11	0.594	0.963
** Crude protein**	65.5	64.3	64.4	0.90	0.268	0.987
**BW, kg**	658	648	649	3.0	0.005	0.764
**BCS, 1-5**	2.30	2.28	2.30	0.012	0.346	0.260

a Monensin (MON); Phytogenic additive (PHY) fed at 89.10 mg/kg DM; blend of phytogenic additives, autolyzed yeast, and organic minerals (BLD) fed at 1.87 g/kg diet DM.

b Orthogonal contrasts: MON vs. PHY and BLD (C1); and PHY vs. BLD (C2).

c No sorting = 1, values < 1 indicates sorting against, and values > 1 indicates sorting for particles on the particular particle size range. Sorting index was calculated according to Silveira et al. (2007).

**Table 3.** Ruminal fermentation parameters in lactating dairy cows fed phytogenic additives alone or combined with autolyzed yeast and organic microminerals (*n* = 12 cows, or 4 cows per treatment).

Item	Treatment^a^			SEM	*P-*value^b^	
	MON	PHY	BLD		C1	C2
**pH**	6.91	6.76	6.93	0.081	0.559	0.182
**NH_3_-N, mg/dL**	9.76	12.0	12.2	1.69	0.284	0.951
**Volatile fatty acids, mol/100 mol**						
**Acetate**	64.6	61.7	64.1	1.31	0.327	0.228
**Propionate**	19.8	19.8	20.1	0.89	0.844	0.781
**Isobutyrate**	0.89	0.93	0.83	0.074	0.930	0.352
**Butyrate**	11.8	13.9	11.8	1.10	0.480	0.198
**Isovalerate**	1.7	1.99	1.66	0.289	0.712	0.431
**Valerate**	1.21	1.61	1.56	0.208	0.191	0.885
**Acetate to propionate ratio**	3.27	3.16	3.21	0.175	0.594	0.797

a Monensin (MON); Phytogenic additive (PHY) fed at 89.10 mg/kg DM; blend of phytogenic additives, autolyzed yeast, and organic minerals (BLD) fed at 1.87 g/kg diet DM.

b Orthogonal contrasts: MON vs. PHY and BLD (C1); and PHY vs. BLD (C2).

Cows fed PHY and BLD produced more 3.5% FCM (35.3 vs. 33.8 kg/d; *P *< 0.05) and ECM (35.7 vs. 34.4 kg/d), with higher milk fat yield (1.28 vs. 1.18 kg/d) and fat percentage (3.88 vs. 3.55%) compared with MON (Table 4). Feed efficiency, expressed per kg milk or FCM, was also higher (*P *< 0.05), and SCS tended to be lower (*P *= 0.066). Within phytogenic treatments, PHY resulted in higher SCS (*P *= 0.026) and tended to increase MUN (*P *= 0.052) relative to BLD.

**Table 4.** Milk yield and composition, and feed efficiency in lactating dairy cows fed phytogenic additives alone or combined with autolyzed yeast and organic microminerals (*n* = 39 cows, or 13 cows per treatment).

Item	Treatment^a^			SEM	*P-*value^b^	
	MON	PHY	BLD		C1	C2
**Yield, kg/d**						
**Milk**	33.6	33.5	33.0	0.49	0.547	0.407
** 3.5% FCM^c^**	33.8	35.3	35.3	0.62	0.016	0.986
** ECM^d^**	34.4	35.7	35.6	0.59	0.047	0.884
**Fat**	1.18	1.28	1.28	0.030	0.002	0.985
**Protein**	1.07	1.08	1.05	0.017	0.891	0.217
**Lactose**	1.61	1.62	1.58	0.026	0.823	0.256
**Composition, %**						
**Fat**	3.55	3.8	3.95	0.099	0.003	0.247
**Protein**	3.19	3.19	3.18	0.014	0.916	0.503
**Lactose**	4.8	4.82	4.79	0.019	0.894	0.366
**Milk urea nitrogen, mg/dL**	7.10	6.99	6.29	0.288	0.134	0.053
**SCS^e^**	3.35	3.27	2.89	0.148	0.066	0.026
**Feed efficiency**						
** Milk yield ÷ DM intake**	1.22	1.27	1.26	0.018	0.044	0.548
** 3.5% FCM ÷ DM intake**	1.24	1.34	1.35	0.023	<0.001	0.835

a Monensin (MON); Phytogenic additive (PHY) fed at 89.10 mg/kg DM; blend of phytogenic additives, autolyzed yeast, and organic minerals (BLD) fed at 1.87 g/kg diet DM.

b Orthogonal contrasts: MON vs. PHY and BLD (C1); and PHY vs. BLD (C2).

c Calculated according to Sklan et al. (1992).

d Calculated according to NRC (2001).

e Somatic cell linear score was calculated according to Shook (1993).

**Fig. 1.**
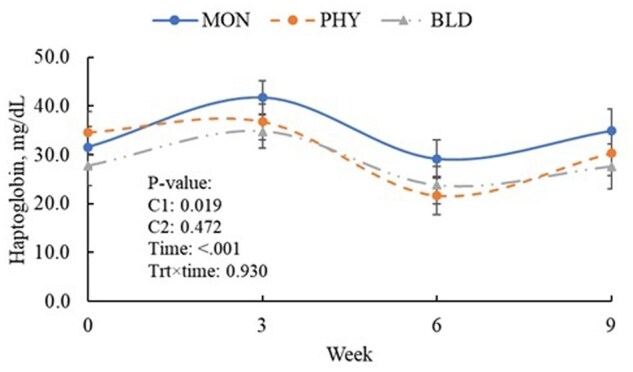
Serum haptoglobin concentrations of in lactating dairy cows fed phytogenic additives alone or combined with autolyzed yeast and organic microminerals. Monensin (MON); phytogenic additive (PHY) fed at 89.10 mg/kg DM; blend of phytogenic additives, autolyzed yeast, and organic minerals (BLD) fed at 1.87 g/kg diet DM. Orthogonal contrasts: MON vs. PHY and BLD (C1); and PHY vs. BLD (C2). Error bars are SE.

Cows fed MON had higher Na, Cl, and urea nitrogen concentrations (*P *< 0.05), lower amylase (*P *= 0.035), and tended to show higher P (*P *= 0.084) and lower K (*P *= 0.088) compared with PHY treatments (Table 5). Among phytogenic additives, PHY cows had higher K (*P *= 0.001) and urea nitrogen (*P *= 0.009), but lower glucose (*P *= 0.016), and tended to have higher vitamin E (P = 0.093), P (*P *= 0.093), and ALT (*P *= 0.070) than BLD. Cows supplemented with PHY or BLD also had lower haptoglobin concentrations than those fed MON (Fig. 1).

**Table 5.** Blood metabolites in lactating dairy cows fed phytogenic additives alone or combined with autolyzed yeast and organic microminerals (n = 39 cows, or 13 cows per treatment).

Item	Treatment^a^			SEM	*P-*value^b^	
	MON	PHY	BLD		C1	C2
**Vit. E, mg/L**	7.31	7.43	6.67	0.310	0.491	0.093
**β-carotene, mg/L**	1.53	1.44	1.55	0.096	0.753	0.398
**Na, mmol/L**	139	138	138	0.3	0.048	0.859
**K, mmol/L**	4.23	4.39	4.22	0.032	0.084	0.001
**Cl, mmol/L**	101	100	99.8	0.338	0.006	0.503
**Ca, mmol/L**	2.64	2.60	2.60	0.035	0.322	0.961
**P, mmol/L**	2.47	2.41	2.27	0.058	0.088	0.093
**TCO_2_, mmol/L**	29.9	30.5	30.9	0.40	0.132	0.557
**Glucose, mg/dL**	65.9	63.6	66.2	0.87	0.247	0.016
**Hematocrit, %PCV**	24.5	24.2	23.7	0.32	0.176	0.288
**pH**	7.47	7.47	7.46	0.007	0.886	0.254
**PCO_2_, mmHg**	39.9	39.9	41.0	0.71	0.502	0.283
**HCO_3_, mmol/L**	28.7	29.3	29.7	0.38	0.123	0.494
**BEECF, mmol/L**	5.05	5.75	5.98	0.426	0.131	0.705
**ANGAP, mmol/L**	13.3	13.1	13.1	0.29	0.533	0.880
**Hemoglobin, g/dL**	7.96	8.00	7.91	0.097	0.992	0.510
**Globulin, g/L**	52.5	50.1	52.7	1.20	0.466	0.131
**Albumin, g/dL**	2.80	2.78	2.73	0.033	0.251	0.291
**Total protein, g/dL**	7.96	7.95	7.92	0.079	0.791	0.821
**Creatinine, mg/dL**	0.72	0.67	0.74	0.043	0.758	0.239
**BUN, mg/dL**	14.5	13.8	11.7	0.50	0.008	0.009
**ALP, U/L**	2.80	2.78	2.73	0.033	0.251	0.291
**ALT, U/L**	21.9	22.4	20.7	0.65	0.709	0.070
**AMY, U/L**	50.0	52.2	53.2	1.01	0.035	0.471
**TBIL, mg/dL**	0.29	0.29	0.30	0.006	0.719	0.449

a Monensin (MON); Phytogenic additive (PHY) fed at 89.10 mg/kg DM; blend of phytogenic additives, autolyzed yeast, and organic minerals (BLD) fed at 1.87 g/kg diet DM.

b Orthogonal contrasts: MON vs. PHY and BLD (C1); and PHY vs. BLD (C2).

TCO_2_, Total blood carbon dioxide; PCO_2_, Partial pressure of carbon dioxide; HCO_3_, Bicarbonate; BEECF, Extracellular base excess; ANGAP, Anion GAP; ALP, Phosphatase alkaline; ALT, Alanine aminotransferase; AMY, Amylase; TBIL, Total Bilirubin.

Cows fed MON consumed more nitrogen (+30 g/d; *P *= 0.050) but converted less into milk protein (23.0% vs. 23.5%; *P *= 0.021) compared with PHY and BLD (Table 6). Within phytogenic treatments, BLD tended to excrete more urinary N (+41 g/d; *P *= 0.094) and showed lower milk N efficiency (23.0% vs. 24.0%; *P *= 0.080) compared with PHY. Purine derivative excretion did not differ among treatments (*P *≥ 0.145).

**Table 6.** Nitrogen utilization and urinary purine derivative excretion in lactating dairy cows fed phytogenic additives alone or combined with autolyzed yeast and organic microminerals (*n* = 39 cows, or 13 cows per treatment).

Item	Treatment^a^			SEM	*P-*value^b^	
	MON	PHY	BLD		C1	C2
**N use, g/d**						
** N intake**	738	704	713	11.9	0.050	0.580
** Urinary N**	228	223	264	19.1	0.459	0.094
** Fecal N**	256	250	255	9.8	0.711	0.704
** Milk N**	168	168	165	2.9	0.758	0.388
** Unaccounted N**	76.1	57.4	39.9	21.76	0.272	0.536
**N use, % N intake**						
** Urinary N**	0.31	0.32	0.37	0.028	0.261	0.179
** Fecal N**	0.34	0.36	0.36	0.009	0.172	0.987
** Milk N**	0.23	0.24	0.23	0.003	0.021	0.080
** Unaccounted**	0.11	0.08	0.04	0.031	0.168	0.366
**Urinary output, L/d**	32.3	31.1	31.9	0.98	0.487	0.535
**Urinary N, %**	0.70	0.73	0.80	0.046	0.215	0.195
**Purine derivatives**						
** Uric acid, mmol/L**	1.23	1.17	1.30	0.127	0.966	0.483
** Allantoin, mmol/L**	13.9	13.6	15.5	1.19	0.649	0.286
** Uric acid, mmol/d**	38.3	35.2	38.6	3.46	0.741	0.496
** Allantoin, mmol/d**	431	410	486	36.0	0.697	0.145

a Monensin (MON); Phytogenic additive (PHY) fed at 89.10 mg/kg DM; blend of phytogenic additives, autolyzed yeast, and organic minerals (BLD) fed at 1.87 g/kg diet DM.

b Orthogonal contrasts: MON vs. PHY and BLD (C1); and PHY vs. BLD (C2).

## Discussion

We hypothesized that supplementing dairy cows with phytogenic additives (PHY and BLD) would enhance milk production and digestive function, leading to superior performance compared with monensin. Furthermore, we expected that the combination of phytogenic compounds with autolyzed yeast and additional organic microminerals would provide a synergistic benefit to cow performance. Indeed, cows receiving phytogenic additives showed improvements in milk fat content and milk fat yield, while maintained a milk yield comparable to cows fed MON. Additionally, phytogenic additives reduced DM and nutrient intake, resulting in higher feed efficiency. Although these performance benefits were hypothesized to result from altered rumen fermentation, our data—based on samples collected once daily using an esophageal probe—did not provide conclusive evidence to confirm this mechanism.

Monensin is widely used in dairy cow nutrition to increase milk production efficiency when fed between 11 to 22 g/t (FDA 2004) and has been used as positive control in previous studies with ruminants (Oh et al. 2018; Repetto et al. 2025). The dose used in this study (17.38 mg/kg DMI) was chosen based on the findings of a recent systematic review (Rezaei Ahvanooei et al. 2023), which identified an optimal dosage of approximately 16 ppm (mg/kg) to enhance feed efficiency and milk yield. For the phytogenic additive we administered 89.10 mg/kg DM, aligning with manufacturer guidelines and supported by studies showing improvements in feed intake and nutrient digestibility at similar dosages in ruminants (Castillo-Lopez et al. 2021; Kholif et al. 2021; Rodrigues et al. 2019). The autolyzed yeast dose of 668.54 mg/kg DM was based on recent studies, indicating its positive effects on ruminal fermentation, feed efficiency (Knollinger et al. 2022; Nasiri et al. 2023; Takiya et al. 2024).

The phytogenic additive used in this study is a formulated blend of spices, extracts, and herbs, including L-menthol, thymol, eugenol, mint oil (*Mentha arvensis*), and clove powder (*Syzygium aromaticum*). Previous research has highlighted several potential benefits of this product, including its influence on rumen fermentation and immune modulation (Castillo-Lopez et al. 2023; Uyarlar et al. 2024). Additionally, Rivera-Chacon et al. (2022) observed a reduced inflammation, while Kröger et al. (2017) reported improvements in ruminal pH, salivation, and rumination during a high-concentrate diet challenge. In lactating dairy cows, PHY supplementation has been associated with increased milk production, improved ECM, and fat content, particularly in older cows (Ramirez et al. 2020). In finishing steers, PHY supplementation has shown a trend toward higher average daily gain (ADG) and larger *longissimus* muscle area (Brand et al. 2019), while growing heifers exhibited numerically higher final BW and ADG compared to control diets (Yang et al. 2023). Additionally, the essential oils and plant extracts in this product have been shown to improve ruminal fermentation and blood parameters, contributing to better post-weaning performance, and serving as a natural alternative to monensin in calves (Akbarian-Tefaghi et al. 2018).

In this study, phytogenic additives did not affect fermentation parameters or apparent total-tract digestibility, providing no clear explanation for the observed decrease in DMI when compared to MON. Similarly, previous research by Rivera-Chacon et al. (2022), reported a tendency for decreased DMI with phytogenic supplementation in non-lactating Holstein cows fed a high-concentrate diet designed to induce subacute ruminal acidosis. However, differences in physiological status, diet composition, and metabolic demands limit the direct comparability of these findings to the current study. In contrast, Jantzi et al. (2021) evaluating PHY in lactating Holstein cows reported an increase in DMI, along with improvements in milk yield, ECM, and a reduced incidence of health disorders. Further research is warranted to elucidate the mechanisms through which phytogenic additives influence DMI and their potential role in improving feed efficiency.

Cow fed phytogenic additives (PHY and BLD) exhibited a distinct sorting behavior compared to the MON group, with cows showing a reduced preference for smaller particles (<4 mm) and an increased tendency to select larger particles (>19 mm) while displaying less selectivity toward particles between 8 and 4 mm when compared to MON. This behavior aligns with previous research showing that bulls supplemented with the same phytogenic additive fed in our trial preferentially selected mid-size particles and demonstrated a tendency to avoid smaller particles (Piran Filho et al. 2021). Interestingly, cows in the PHY group tended to avoid particles between 19 and 8 mm compared to those in BLD group, suggesting that phytogenic additives alone may influence particle selection differently from the combination treatment. This shift in sorting behavior could reflect the capacity of phytogenic additives to alter feeding patterns, potentially enhancing nutrient intake efficiency by modulating satiety or rumen conditions (Allen 2020), ultimately supporting a more balanced intake across particle sizes and promoting steadier rumen fermentation.

Supplementation with phytogenic additives increased milk fat content and yield, as well as 3.5%FCM and ECM. These findings align with previous research demonstrating that phytogenic additives can positively impact milk yield and total solid content (Janzi et al. 2021; Kholif et al. 2021), FCM, and ECM (Rodrigues et al. 2019). Although these benefits are often linked to enhanced ruminal fermentation (Busquet et al. 2005), no clear evidence for such effects was observed in this study. These inconsistencies may be attributed to limitations in the sampling methodology, as well as variations in sample size, phytochemical composition, and the timing of rumen collections relative to feeding. Consistent with these observations, no differences in DM and nutrient digestibility were detected between treatments, in agreement with Benchaar et al. (2006). Because no differences were observed in apparent total-tract nutrient digestibility or ruminal fermentation, the positive effects of phytogenic additives on ECM and fat yields are likely linked to their influence on nitrogen metabolism or inflammation. Cows receiving phytogenic additives may have benefited from reduced energetic costs associated with nitrogen metabolism. By lowering CP intake without compromising digestibility, these cows likely reduced the need for deamination and urea synthesis, thereby sparing energy that could be redirected toward other productive functions (Reed et al. 2017). Moreover, excessive protein intake and inefficient nitrogen utilization are often associated with elevated circulating urea and inflammatory responses, reflected by higher concentrations of acute-phase proteins such as haptoglobin (Lopreiato et al. 2020). In the present study, the reduced blood urea nitrogen concentration in cows fed phytogenic additives compared with MON supports the interpretation of improved nitrogen metabolism. Haptoglobin is an acute-phase protein widely recognized as a biomarker of inflammation in dairy cows. Its synthesis is stimulated by proinflammatory cytokines, such as interleukin-1 and tumor necrosis factor-α, during activation of the innate immune system. Elevated circulating haptoglobin concentrations therefore indicate systemic inflammation and an activated immune system, which are associated with increased metabolic and energetic costs that can impair productivity (Eckersall and Bell 2010; Kvidera et al. 2017). Although cows fed BLD had lower blood urea nitrogen and MUN concentrations compared with those fed PHY, milk yield and composition were similar between the two groups, suggesting that the improvements in nitrogen metabolism with BLD were not sufficient to further enhance cow performance. Autolyzed yeast provides peptides and nucleotides that enhance rumen microbial growth and microbial protein synthesis, while organic microminerals support enzymes involved in amino acid metabolism and liver function, improving nitrogen utilization (Duplessis and Royer 2023; Rabiee et al. 2010; Takiya et al. 2024).

The improvements in performance and efficiency may also be attributed to the effect of phytogenic compounds in reducing nitrogen intake while enhancing nitrogen utilization in milk, which aligns with the findings of Takiya et al. (2023). Although no differences were observed in nitrogen excretion in urine and feces in our study, the blood metabolite data support the positive effects of phytogenic additives in reducing nitrogen intake. Urine output in this study was estimated from creatinine excretion, a practical and commonly used approach in ruminant research, but not the most reliable. Creatinine excretion can vary with BW, muscle mass, diet, and physiological status, and the assumption of constant excretion per unit of BW may not always hold, particularly under dietary treatments that affect protein or energy metabolism (Chizzotti et al. 2008; Reynolds and Kristensen 2008). Thus, while this method provided useful relative comparisons among treatments, the results should be interpreted with caution.

Studies have indicated that phytogenic additives can modulate ruminal fermentation patterns and nutrient digestibility (Castillo-Lopez et al. 2022; Rivera-Chacon et al. 2022). For example, Rivera-Chacón et al. (2022) observed a tendency for reduced propionate concentrations without changes in total VFA, whereas Castillo-López et al. (2022) reported higher butyrate levels, a trend toward increased total VFA, and enhanced rumen degradation of corn when supplementing a similar phytogenic composition compared with a control group (without MON). Moreover, Kholif et al. (2021) and Takiya et al. (2023) found enhanced nutrient digestibility with phytogenic supplementation compared to control. Taken together, these findings suggest that although these additives can influence fermentation, their overall impact on nutrient utilization efficiency are variable and likely dependent on additive dose and composition, and diet composition.

While MON enhances propionate availability for gluconeogenesis, thereby increasing plasma glucose levels (Duffield et al. 2008), this shift in fermentation patterns may also promote gram-negative bacterial growth and elevate lipopolysaccharide levels triggering inflammation (Johnzon et al. 2018; Schären et al. 2017; Weimer et al. 2008). Additionally, Knollinger et al. (2022) found that yeast-derived compounds, similar to those used in this study, decreased albumin while increasing serum amyloid A, further suggesting an inflammatory response. Inflammation has been linked to elevated glucose concentrations (Qiao et al. 2024), which could explain why glucose levels were lower in the PHY group compared to MON and BLD. However, MON was associated with higher haptoglobin levels than both PHY and BLD treatments, which suggests that MON may induce a more pronounced inflammatory response compared to the other treatments. This discrepancy in inflammatory response could also vary depending on the stage of lactation, as yeast supplementation is often associated with reduced inflammation due to rumen stabilization. Although globulin and albumin values did not differ significantly between treatments, their trends align with inflammation-related metabolic responses. Specifically, the PHY group exhibited lower globulin levels compared to the other treatments and higher albumin levels compared to the BLD treatment. Inflammatory responses typically result in increased globulin and decreased albumin concentrations, reflecting the liver’s shift toward acute-phase protein production (Cattaneo et al. 2021). While these effects appear relatively small, their long-term impact on dairy cow health and productivity remains unclear. Further research is needed to determine whether prolonged exposure to different feed additives could lead to more pronounced metabolic and immune adaptations.

When comparing cows supplemented exclusively with PHY to those receiving BLD, the PHY group demonstrated improved nitrogen partitioning into milk rather than urine, suggesting a potential environmental benefit by reducing nitrogen waste. However, the PHY group also exhibited higher SCS and MUN levels compared to the BLD group. The reduction in SCS observed in the BLD group may be attributed to yeast supplementation, as previous studies in transition dairy cows have linked yeast to improved inflammatory and immune responses (Cattaneo et al. 2023). Reduced MUN levels in the BLD group, closely linked to protein intake and metabolic efficiency (Roseler et al. 1993), may also reflect differences in protein metabolism between the two supplementation strategies.

The elevated serum Na and Cl concentrations in the MON group can be attributed to the ionophore activity of MON, which forms lipophilic complexes with cations and facilitates their transmembrane transport, with a strong preference for sodium (Martineau et al. 2007; Pressman et al. 1980). In contrast, the elevated serum K levels in the PHY group suggest improved electrolyte balance, potentially linked to enhanced nutrient absorption or alterations in ruminal microflora, as reported by Takiya et al. (2023). Moreover, the trend toward higher serum concentrations of vitamin E and phosphorus associated with the decrease in glucose levels in the PHY compared to BLD group suggests that phytogenic additives alone may be more effective at enhancing antioxidant capacity and mineral bioavailability. This finding implies that the inclusion of autolyzed yeast and organic minerals may modulate, rather than amplify, the effects of phytogenic additives, highlighting the unique role of phytogenics in optimizing mineral metabolism.

Acute phase proteins, such as haptoglobin and fibrinogen are secreted in response to immune challenges such as infections, inflammation, and metabolic disturbances (Saco and Bassols 2023). In cows, these proteins are sensitive markers of inflammatory processes and can help detect subclinical conditions, particularly mastitis (Nielsen et al. 2004). Our study found higher serum haptoglobin and a tendency for higher SCS levels in the MON group than in the other treatment groups, supporting the ability of phytogenic additives in immune modulation. These results are consistent with previous studies showing that phytogenic supplementation reduces haptoglobin and histamine concentrations in cattle, thereby contributing to lower systemic inflammation (Humer et al. 2018; Rivera-Chacon et al. 2022). Similar plasma concentrations of protein and energy metabolism and hepatic biomarkers, minerals, and enzymes across treatment groups within the physiological range (Cozzi et al. 2011) indicated that neither MON nor phytogenic additives negatively affect liver function or the overall health status (Russell and Roussel 2007). Together, these findings showed that phytogenic additives may enhance immune resilience and are safe for liver health in dairy cows.

## Conclusion

Phytogenic additives, whether used alone or in combination with autolyzed yeast and additional organic microminerals achieved better performance than MON in dairy cow diets. They support comparable or enhanced milk production and composition and improve nitrogen metabolism. Additionally, data showed a potential reduction in inflammatory processes.

## Data Availability

The datasets generated and/or analyzed during the current study are available from the corresponding author on reasonable request.
